# Friendship influence moderating the effect of a web-based smoking prevention program on intention to smoke and knowledge among adolescents

**DOI:** 10.1016/j.abrep.2020.100335

**Published:** 2020-12-30

**Authors:** Georges E. Khalil, Alexander V. Prokhorov

**Affiliations:** The University of Florida, College of Medicine, United States; The University of Texas MD Anderson Cancer Center, United States

**Keywords:** Adolescent, Smoking, Tobacco, Influence, Prevention

## Abstract

**Purpose:**

Web-based tobacco prevention programs for adolescents have stressed human-computer interaction, but they have not yet extensively applied social interactivity (i.e., computer-mediated or face-to-face interactions). This study examines if prior tendencies for positive social influence (PSI), negative social influence (NSI), and having friends who smoke (HFS) moderate the success of a web-based program for smoking prevention.

**Methods:**

Participants were 101 adolescents (aged 12–18 years) from the ASPIRE-Reactions study, a randomized controlled trial comparing a program called ASPIRE with its text-based version. Knowledge of tobacco consequences and intention to smoke were assessed at baseline and end-of-treatment. Tendency for PSI (i.e., avoid tobacco when advised by friends) and NSI (i.e., accept tobacco when offered by friends) were measured at baseline. Repeated-measures mixed-effect modeling was used for hypothesis-testing.

**Results:**

While controlling for ASPIRE effects, both NSI and PSI predicted lower intention to smoke. Adolescents with high NSI were more likely to show a group difference with respect to change in intention to smoke, but not knowledge. Although not significant, this moderation effect was observed in the expected direction with PSI, predicting intention to smoke and knowledge. HFS significantly moderated the effect of ASPIRE on knowledge. Associations with depression and internet use are also described.

**Conclusion:**

The results suggest that adolescents with high tendencies for NSI may particularly benefit from web-based interventions such as ASPIRE. Also, web-based interventions may benefit from peer-to-peer interactions, boosting PSI. While current web-based programs include human-computer interaction as their main feature, this study suggests considering social interactivity.

## Highlights

• ASPIRE can reduce intention to smoke with adolescents who have a tendency for NSI.
• PSI tendency predicts intention to smoke and knowledge, despite ASPIRE effect.
• ASPIRE can improve tobacco knowledge among adolescents who have friends who smoke.
• Adolescents exposed to friends who smoke may benefit from programs that promote PSI.

## 1 Introduction

In the United States, more than 27% of high school students report being current tobacco users (Gentzke et al., 2019). While electronic cigarettes (e-cigarettes) have reached highest rates (20.8%), current use of combustible products remains a public health problem, including cigarettes (8.1%), cigars (7.6%), smokeless tobacco (5.9%), and hookahs (4.1%) (Gentzke et al., 2019). Tobacco smoking is initiated during this critical age period, leading to early nicotine dependence (Bahelah et al., 2018, Case et al., 2018), early carcinogenic processes (Popa, 2012), and early signs of pulmonary and cardiovascular diseases (Gold et al., 1996, Perrine et al., 2019). The plethora of medical and psychological outcomes of combustible tobacco use at an early age highlight the importance of preventing initiation during adolescence.

Owing to the rise in interactive online features, web-based programs have become an increasingly popular method to communicate tobacco risks to adolescents (Lustria et al., 2013, Thomas et al., 2015). By interacting with avatars, navigating virtual environments, and receiving health information through entertaining animations, adolescents can become emotionally involved in the content and provoked to think deeper about their experience (Khalil et al., 2017). Moreover, web-based programs can be tailored to provide content based on gender, culture, and stage of tobacco acquisition, and they offer improved access to tobacco prevention services (Hollis et al., 2005, Redding et al., 2014).

Despite such features, computer-based programs for tobacco prevention that address social influence through social interactivity (i.e., computer-mediated or face-to-face interactions) have not yet been designed (Thomas et al., 2015). An example is a web-based intervention called A Smoking Prevention Interactive Experience (ASPIRE). The program includes a series of videos and activities that promote a tobacco-free lifestyle (Prokhorov et al., 2010), and it has shown considerable promise in reducing smoking initiation after an 18-month follow-up (Prokhorov et al., 2008).

Despite the overall effectiveness of ASPIRE, the lack of social influence features may affect the success of this program. Social theoretical models such as the social learning theory (Bandura, 1977) explain that adolescent smoking is acquired through social interactions with peers who encourage substance use (Petraitis, Flay, & Miller, 1995). This result has been mainly attributed to having friends who smoke (HFS) (Bountress, Chassin, Presson, & Jackson, 2016). Additional evidence indicates that a tendency to be influenced by friends can predict tobacco use among adolescents. A tendency to accept tobacco when offered by peers has been shown to predict tobacco initiation (Pierce, Choi, Gilpin, Farkas, & Merritt, 1996) and forms a direct indicator of negative social influence (NSI). While HFS and NSI may promote tobacco use, research has indicated that positive social influence (PSI) through a tendency to avoid tobacco when advised by a social circle may act as a preventative factor (Kalesan et al., 2006, Houle et al., 2017, Coulombe et al., 2019, Van Ryzin and Roseth, 2018). Accordingly, both positive and negative influence are potential predictors of tobacco use that deserve attention.

Considering that computer-based programs such as ASPIRE lack social interaction features, little is still known regarding the process by which positive and negative influence are associated with program effectiveness. The objective of the present study was to examine whether indicators of social influence through friendships (HFS, NSI, and PSI) can affect the success of ASPIRE. Specifically, we hypothesized that: (1) NSI tendencies weaken the ability of ASPIRE to decrease intention to smoke and improve knowledge; (2) PSI tendencies strengthen the ability of ASPIRE to decrease intention to smoke and improve knowledge; (3) NSI tendencies have a stronger effect on ASPIRE’s success than PSI; and (4) HFS weakens the ability of ASPIRE to decrease intention to smoke and improve knowledge.

## 2 Materials and methods

### 2.1 Study design

This study was conducted under the randomized controlled trial ASPIRE-Reactions (N = 101), with assessments conducted three days prior to treatment and by the end of a one-month-long treatment (Khalil et al., 2017). The trial was registered at the Clinical Trials registry, NCT02469779 (Eysenbach, 2012, Eysenbach and Group, 2011).

### 2.2 Participant recruitment

Recruitment procedures have been previously described by Khalil and colleagues (Khalil et al., 2017). Briefly, four after-school programs in Houston, Texas were randomly selected for recruitment, including the Boys and Girls Clubs and the Young Men's Christian Association (YMCA). We announced the study to 509 adolescents, and 110 agreed to participate. A total of 101 adolescents were eligible (i.e., were 12–18 years of age, and were nonsmokers, that is they have not smoked in the past year, not even one cigarette, cigar, or hookah). Participants provided consent and obtained parental permission. The Institutional Review Board approved this study. At the end of the trial, 98 adolescents (97.03%) had completed all survey data.

### 2.3 ASPIRE and control groups

Adolescents in the ASPIRE group received the program in its complete format. ASPIRE features interactivity and entertainment that present with cartoon animations, testimonies from other adolescents, and educational activities. With respect to content, ASPIRE development was guided by the *trans*-theoretical model of the stages of change (Prokhorov et al., 2010). The model explains that adolescents move from a stage of pre-contemplation to contemplation, action, and then maintenance of healthy behavior, with processes of change facilitating change in stages. Adolescents in the control group received the same health information presented in ASPIRE on a computer screen, but without any features of interactivity or entertainment. Under both conditions, adolescents were not presented with features of social interactivity (i.e., peer-to-peer interaction). Adolescents received the intervention once a week for five weeks (Khalil et al., 2017).

### 2.4 Study measures and assessment

The survey measures have been previously tested and validated prior to implementation (e.g., Fujimoto and Valente, 2012, Pierce et al., 1996, Straub et al., 2003). At baseline, we collected information on age, gender, race/ethnicity, and number of hours of internet use per week. Depression was assessed using the Center for Epidemiologic Studies Depression Scale for Children (CES-DC) (Shahid, Wilkinson, Marcu, & Shapiro, 2011). At baseline and follow-up, we measured intention to smoke using three questions such as “Do you think in the future you might try a cigarette, cigar, or hookah?” (Pierce et al., 1996, Straub et al., 2003, Vitória et al., 2011, Patiño-Masó et al., 2019). On a 5-point Likert scale, answer choices ranging from “definitely yes” to “definitely not” (Cronbach’s alpha = 0.80). A continuous variable allowed us to keep information about where adolescents stand in their level of intention. We measured knowledge of tobacco consequences by asking participants to identify tobacco use effects, using 21 items such as “heart problems” and “fever”, with answer choices “yes”, “no”, and “I am not sure/I do not know” (Cronbach’s alpha = 0.76) (Anjum, Ahmed, & Ashfaq, 2008).

At baseline, we assessed NSI and PSI through an ego-network assessment questionnaire (Fujimoto & Valente, 2012). By using three items, we asked adolescents to report the likelihood of accepting a cigarette, cigar, or hookah from each of their three best friends (3 items, Cronbach’s alpha = 0.87). Similarly, we assessed baseline PSI with three items asking about the likelihood of avoiding a cigarette, cigar, or hookah when advised by each of the three best friends (Cronbach’s alpha = 0.96). Answer choices for both measures included a 5-point Likert scale, from “not at all likely” to “extremely likely.” At baseline, we assessed the number of friends who smoke by asking: one “How many of your friends smoke?” (Gerrard, Gibbons, Benthin, & Hessling, 1996):

### 2.5 Statistical analysis

Analyses were conducted using STATA14.0 (Stata Corp LP). We tested relationships between participant characteristics and social influence measures using one-way analyses of variance and Chi-square statistics. Baseline relationships between social influence measures and outcomes (intention to smoke and knowledge) were examined using multiple regression models. With the use of repeated-measures mixed effect models, NSI, PSI, and HFS were examined as predictors of intention to smoke and knowledge, controlling for ASPIRE effect and other identified covariates.

For moderation analysis, we opted to dichotomize the main predictors (i.e., based on the median for NSI and PSI, and on having or not having friends who smoke for HFS). This was performed to effectively interpret the results of three-way interaction effects (Le & Johnson, 2008). To test the moderating role of NSI, we conducted a series of repeated-measures mixed-effects models that predicted intention to smoke and another series of models that predicted knowledge. The first pair of models examined the interaction term [group (ASPIRE versus control) × time (baseline to end of treatment)], testing group differences over time with respect to (1) intention to smoke and then (2) knowledge. The second pair of models included NSI as a covariate, controlling for the group-by-time effect. The third pair of models examined a 3-way moderation effect with 2 (group) × 2 (time) × 2 (baseline NSI) that predicted (1) intention to smoke and (2) knowledge. The same models were conducted to test the moderating effect of PSI tendencies and HFS. Depression level was found to be related to intention to smoke and was included as a covariate in appropriate models. For all models, multicollinearity was tested, and the Huber-White sandwich estimator was used to correct variance estimates for heteroskedasticity. This satisfies non-normal distribution of outcomes by providing appropriate estimates of standard errors. For each finding, unstandardized coefficients were computed with their standard errors and *p-*values.

## 3 Results

### 3.1 Participant characteristics

The average age in this sample (n = 101) was 13.44 (SD = 1.42) years. The majority of participants were Black or African American (41.58%) and non-White Hispanic or Latino (43.56%), and 43.56% were female. Approximately 25% of participants scored above the median on NSI, 62% scored above the median on PSI, and 45% reported having at least one friend who smokes. At baseline, intention to smoke (M = 1.50, SD = 0.68) had a skewness of 1.38, and knowledge (M = 13.49, SD = 3.94) had a skewness of −1.08.

### 3.2 Social influence and demographic characteristics

There was a significant difference between low and high NSI with respect to the number of internet hours per week [F(1, 96) = 6.96, *p* = 0.009] and depression [F(1, 98) = 7.16, *p* = 0.009]. NSI did not correlate with age [F(1, 99) = 0.45, *p* = 0.50], gender [χ^2^(1) = 0.01, *p* = 0.92], race [χ^2^(5) = 3.28, *p* = 0.657], or number of friends who smoke [F(1, 94) = 1.26, *p* = 0.26].

On the other hand, PSI was not related to the number of internet hours per week [F(1, 96) = 2.36, *p* = 0.13], age [F(1, 99) = 0.35, *p* = 0.56], gender [χ^2^(1) = 0.56, *p* = 0.45], race [χ^2^(5) = 6.87, *p* = 0.23], or depression [F(1, 98) = 0.14, *p* = 0.71]. PSI was, however, significantly related to a lower number of friends who smoke [F(1, 94) = 4.87, *p* = 0.03]. Finally, a higher number of friends who smoke was significantly related to a higher level of depression (*r* = 0.24, *p* = 0.02); however, it was not related to age (*r* = −0.03, *p* = 0.80), gender [F(1, 94) = 0.47, *p* = 0.49], or ethnicity [F(5, 89) = 1.15, *p* = 0.34].

### 3.3 Baseline relationships between social influence and outcomes

Baseline data revealed that adolescents with high NSI scored significantly higher on intention to smoke (β = 0.64, *p* < 0.001) and lower on knowledge (β = −0.24, *p* = 0.02), compared with low-NSI adolescents. Conversely, high-PSI adolescents scored significantly lower on intention to smoke (β = −0.35, *p* = 0.001), compared with low-PSI adolescents. The relationship between PSI and knowledge was not significant (β = 0.20, *p* = 0.06).

**Fig. 1:**
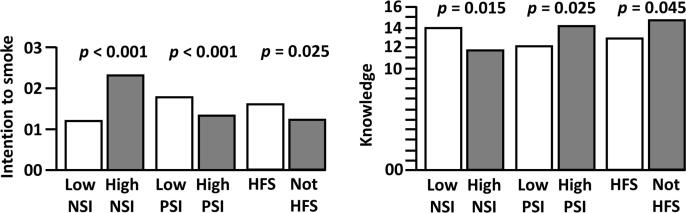
Differences between low and high social influence measures with respect to intention to smoke and knowledge at baseline. PSI stands for positive social influence; NSI stands for negative social influence; HFS stands for having friends who smoke. All p-values are based on one-way analyses of variance.

Based on these results, Fig. 1 presents the differences between low and high social influence measures with respect to intention to smoke and knowledge at baseline. The number of friends who smoke was not related to either intention to smoke or knowledge. However, HFS was significantly related to a higher intention [F(1, 93) = 5.16, *p* = 0.02] and knowledge [F(1, 94) = 4.13, *p* = 0.04].

When both PSI and NSI were added to the model, only NSI presented a significant relationship with intention to smoke (for NSI, β = 0.88, *p* < 0.001; for PSI, β = −0.16*, p* = 0.17). Conversely, neither of the measures was significantly related to knowledge (for NSI, β = −1.84, *p* = 0.05; for PSI, β = 0.13, *p* = 0.84).

### 3.4 Social influence as a predictor of outcomes

Adolescents receiving ASPIRE were more likely to experience a decrease in intention to smoke over time, as compared with those receiving the control intervention (group-by-time effect, B = −0.26, *p* = 0.02). When NSI was added to the model, a significant positive relationship with intention to smoke was observed (B = 0.90, *p* < 0.001). Moreover, when PSI was added to the model, it exhibited a significant negative relationship with intention to smoke (B = −0.455, *p* < 0.001). HFS was not related to intention to smoke (B = 0.22, *p* = 0.14).

Adolescents receiving ASPIRE were more likely to improve in knowledge regarding smoking consequences, when compared with those receiving the control intervention (group-by-time effect, B = 5.32, *p* < 0.001). When added to the model, NSI (B = −1.22, *p* = 0.16), PSI (B = 0.98, *p* = 0.23), and HFS (B = −0.93, *p* = 0.25) were not significantly related to knowledge.

### 3.5 Social influence as a moderator of outcomes

According to moderation analysis of negative influence (Table 1), the three-way interaction between time, conditions, and NSI significantly predicted intention to smoke (B = −1.07, *p* < 0.001). When compared to the control, ASPIRE resulted in significantly lower intention to smoke over time for participants with high NSI (Appendix A). This group difference over time was not observed for participants with low NSI. In a second analysis, there was no significant three-way interaction effect between time, conditions, and NSI when predicting knowledge (B = −3.89, *p* = 0.12).

**Table 1:** Baseline NSI as a moderator of ASPIRE effect on intention to smoke and knowledge.

	Intention to smoke		Knowledge	
	B (*SE*)	*p*	B (*SE*)	*p*
Group				
ASPIRE	−0.06 (0.09)	0.513	0.56 (0.71)	0.431
Control (ref.)				
Time				
End-of-treatment	−0.03 (0.06)	0.668	−4.26 (1.10)	<0.001
Baseline (ref.)				
Group × Time	−0.07 (0.09)	0.388	6.15 (1.26)	<0.001
Baseline NSI				
High	0.87 (0.24)	<0.001	−2.02 (1.38)	0.141
Low (ref.)				
Time × Baseline NSI	0.25 (0.16)	0.125	2.87 (1.93)	0.137
Group × Baseline NSI	0.17 (0.37)	0.634	0.96 (1.74)	0.580
Time × Group × Baseline NSI	−1.07 (0.30)	<0.001	−3.89 (2.51)	0.120
Depressive symptoms	0.05 (0.09)	0.577	−0.68 (0.62)	0.270
Number of friends who smoke	0.02 (0.02)	0.181	−0.24 (0.12)	0.047
Intercept	1.15 (0.14)	<0.001	15.75 (1.27)	<0.001
Wald Chi^2^	87.47	<0.001	68.29	<0.001

*Note.* Two Repeated-Measures Mixed Effect Models. Intention to smoke and knowledge were assessed 3 days before treatment and by the end of a one-month-long treatment (i.e., 33 days post-baseline assessment). ASPIRE stands for a smoking prevention interactive experience. PSI stands for positive social influence. SE stands for standard error.

An analysis of positive influence (Table 2) showed no significant three-way interaction effect between time, conditions, and PSI, predicting intention to smoke (B = 0.41, *p* = 0.08). Also, a three-way interaction effect between time, conditions, and PSI did not significantly predict knowledge (B = 3.59, *p* = 0.09). Having higher PSI remained significantly related to lower intention to smoke over time (B = −0.53, *p* = 0.02). When compared to the control, ASPIRE resulted in lower intention to smoke and higher knowledge over time for participants with low PSI (Appendix B). The results also indicated a significant relationship between depressive symptoms and intention to smoke (B = 0.20, *p* = 0.01).

**Table 2:** Baseline PSI as a moderator of ASPIRE effect on intention to smoke and knowledge.

	Intention to smoke		Knowledge	
	B (*SE*)	*p*	B (*SE*)	*p*
Group				
ASPIRE	−0.15 (0.30)	0.620	2.17 (1.34)	0.105
Control (ref.)				
Time				
End-of-treatment	−0.06 (0.06)	0.664	−1.64 (1.32)	0.213
Baseline (ref.)				
Group × Time	−0.57 (0.21)	0.006	2.83 (1.62)	0.080
Baseline PSI				
High	−0.53 (0.23)	0.024	1.78 (1.23)	0.150
Low (ref.)				
Time × Baseline PSI	−0.02 (0.15)	0.864	−2.84 (1.79)	0.113
Group × Baseline PSI	0.13 (0.32)	0.684	−2.02 (1.50)	0.178
Time × Group × Baseline PSI	0.41 (0.23)	0.080	3.59 (2.15)	0.096
Depressive symptoms	0.20 (0.08)	0.011	−0.84 (0.61)	0.172
Number of friends who smoke	0.01 (0.03)	0.623	−0.23 (0.12)	0.052
Intercept	1.45 (0.24)	<0.001	14.33 (1.62)	<0.001
Wald Chi^2^	35.34	<0.001	53.96	<0.001

*Note.* Two Repeated-Measures Mixed Effect Models. Intention to smoke and knowledge were assessed 3 days before treatment and by the end of a one-month-long treatment (i.e., 33 days post-baseline assessment). ASPIRE stands for a smoking prevention interactive experience. PSI stands for positive social influence. SE stands for standard error.

A third analysis for HFS (Table 3) showed no significant interaction effect between time, conditions, and HFS when predicting intention to smoke (B = −0.15, *p* = 0.51). On the other hand, HFS significantly moderated the effect of ASPIRE on knowledge (B = 5.52, *p* = 0.01). ASPIRE resulted in higher knowledge over time for participants with one or more friends who smoke, compared to participants who had no friends who smoke (Appendix C).

**Table 3:** HFS as a moderator of ASPIRE effect on intention to smoke and knowledge.

	Intention to smoke		Knowledge	
	B (*SE*)	*p*	B (*SE*)	*p*
Group				
ASPIRE	−0.23 (0.16)	0.887	0.53 (0.97)	0.114
Control (ref.)				
Time				
End-of-treatment	0.03 (0.06)	0.646	−1.90 (1.33)	0.151
Baseline (ref.)				
Group × Time	−0.26 (0.12)	0.029	2.68 (1.54)	0.082
Baseline HFS				
Yes	0.25 (0.20)	0.219	−0.15 (1.10)	0.890
No (ref.)				
Time × Baseline HFS	0.04 (0.13)	0.736	−2.89 (1.82)	0.111
Group × Baseline HFS	−0.04 (0.29)	0.901	−0.87 (1.44)	0.547
Time × Group × Baseline HFS	−0.15 (0.22)	0.510	5.07 (2.12)	0.017
Depressive symptoms	0.17 (0.09)	0.069	−0.87 (0.63)	0.169
Intercept	1.07 (0.18)	<0.001	14.97 (1.42)	<0.001
Wald Chi^2^	38.00	<0.001	70.28	<0.001

*Note.* Two Repeated-Measures Mixed Effect Models. Intention to smoke and knowledge were assessed 3 days before treatment and by the end of a one-month-long treatment (i.e., 33 days post-baseline assessment). ASPIRE stands for a smoking prevention interactive experience; HFS stands for having friends who smoke. SE stands for standard error.

## 4 Discussion

While decision-making during adolescence is marked by the power of social influence, little is known regarding how social influence tendencies affect the success of stand-alone digital programs for tobacco prevention. This study provides an initial investigation of the moderating roles of three main social influence indicators (HFS, NSI, and PSI) on the effectiveness of the program ASPIRE.

First, while controlling for the effect of ASPIRE, both NSI and PSI predicted intention to smoke, but they did not predict knowledge. This may be due to the manner by which adolescents process information when making a decision that is based on social influence. The elaboration likelihood model suggests that individuals, particularly adolescents, tend to make decisions by to the message sender (i.e., peripheral processing) rather than paying attention to the content of the message (i.e., central processing) (Petty, Cacioppo, & Kasmer, 2015). As a result, adolescents tend to be influenced by their friends regardless of their knowledge about tobacco. Influence from friends leads adolescents to directly make a decision regarding tobacco, but it does not improve their knowledge. Moreover, from the perspective of social and ecological models (Hovell, Wahlgren, & Adams, 2009), adolescents’ interactions with peers tend to shift perceived norms and support social assimilation rather than information-recall or knowledge-gain (Petraitis et al., 1995). Interestingly, HFS did not predict intention to smoke or knowledge, highlighting the limitation of studies that only consider exposure to smokers as a proxy of social influence, when predicting tobacco outcomes. According to the present results, measuring the tendency of adolescents to accept or reject tobacco from friends is key to capturing social influence because it goes beyond mere exposure and assesses their personal decision to be influenced.

In this study, the success of ASPIRE was directly affected by social influence indictors. The results suggest that adolescents with a tendency for negative influence may be more likely to benefit from ASPIRE than adolescents with low NSI. This may be due to ASPIRE’s effective presentation of information related to the importance of rejecting tobacco when offered by friends. Although using human-computer interaction, ASPIRE’s content includes activities that allow adolescents to practice social skills (Prokhorov et al., 2010). It is hence well tailored to adolescents who have a predisposition to accept tobacco when offered by friends. Unlike NSI or PSI, HFS moderated the effect of ASPIRE on knowledge but not on intention to smoke. In particular, adolescents with friends who smoke were more likely to improve in knowledge about tobacco effects as a result of ASPIRE, when compared with those who do not have friends who smoke. Through social cohesion, adolescents’ high exposure to smokers may have created a social bubble that prevents them from receiving outside information about tobacco (Valente, 2007). As a result of their exposure to ASPIRE, this bubble may burst, allowing them to significantly improve in knowledge.

Although non-central to this study, our analyses identified specific adolescent groups characterized by social influence tendencies. For instance, adolescent nonsmokers who tend to accept tobacco when offered by friends were more likely to exhibit depressive symptoms and spend more time on the internet. These results confirm the findings of previous literature (Cruwys et al., 2014, Elhai et al., 2018, Sanders et al., 2000). In addition, as supported by previous work, HFS was significantly related to a higher depression level (Dingle et al., 2015, Liu et al., 2017, Costello et al., 2019). PSI was related to a lower number of friends who smoke. This suggests that encouraging positive influence may shift the quality of friendships and lower exposure to smokers. This strategy has been the main goal of some interventions for adolescents (Thomas et al., 2013, Valente, 2012).

Some study limitations must be noted. First, we did not consider adolescents’ position in their friendship networks, which may have led to the formation of friendships with smokers. Nevertheless, the study concentrated on immediate friendships. Second, higher levels of influence (e.g., at the parental or organizational levels) may have played a crucial role in predicting the success of the ASPIRE program. However, it must be noted that the results of our models did not change when controlling for parental smoking status. The next line of research may examine multi-level factors of social influence that can be crucial to preventing tobacco use. While the trial did not examine long-term outcomes, intention to smoke has been shown to be the most potent predictor of tobacco initiation among adolescents (Pierce et al., 1996). This was determined with the dichotomous form of intention termed susceptibility to smoke. With a larger sample, future research may confirm the current findings with susceptibility to smoke. In such a larger study, correcting for multiple tests would be warranted. Nevertheless, as a continuous variable, “intention to smoke” provides richer information about where adolescents stand regarding their level of intention. Finally, in this study, e-cigarette use was not examined. Although vaping is currently the most prevalent form of tobacco use, ASPIRE was originally designed to communicate about combustible tobacco products. Nevertheless, future research may consider examining the current social influence pathways in the context of e-cigarette use, particularly with the newer version of ASPIRE that will include messages about all tobacco products.

In practice, our findings propose that adolescents, particularly those exposed to friends who smoke, may benefit from tobacco control programs that extend beyond human-computer interaction, by allowing social interaction. Given the increasingly important role of social connection during adolescents’ development (Bagwell & Bukowski, 2018), peer-to-peer interaction may improve tobacco prevention intervention response by promoting positive influence. For future research, our plan is to examine these social influence factors as moderators of a program’s success in preventing smoking behavior in the long-term and consider the study results in the context of vaping prevention. As part of such an examination, we will assess these factors over time as they evolve based on adolescents’ networks of friends. Moreover, future work may consider examining processes of adolescent social influence in the context of new and emerging products (e.g., electronic-cigarettes).

## Author disclosure

### Role of funding sources

Research reported in this publication was supported by the National Cancer Institute of the National Institutes of Health (R25 CA057730; Principal Investigator: Shine Chang) and by the National Institute on Drug Abuse of the National Institutes of Health (K99 DA044277; Principal Investigator: Georges Khalil). The content is solely the responsibility of the authors and does not necessarily represent the official views of the National Institutes of Health.

### Contributors

Georges E. Khalil is responsible for the design of the study; Alexander V. Prokhorov provided guidance on the design of the study; Georges E. Khalil was responsible for the data analysis; Georges E. Khalil, and Alexander V. Prokhorov contributed to the conceptualization and design of the paper; Georges E. Khalil drafted the paper; Alexander V. Prokhorov critically revised the paper. Both authors read and approved the final version. Georges E. Khalil had full access to all of the data in the study and takes responsibility for the integrity of the data and the accuracy of the data analysis.

## CRediT authorship contribution statement

**Georges E. Khalil:** Conceptualization, Data curation, Formal analysis, Writing - original draft, Writing - review & editing. **Alexander V. Prokhorov:** Visualization, Validation, Writing - review & editing.

## Declaration of Competing Interest

All authors declare that they have no conflicts of interest.
