# Supplementary material for: Friendship influence moderating the effect of a web-based smoking prevention program on intention to smoke and knowledge among adolescents
Source: Addict Behav Rep. 2020 Dec 30;13:100335. doi: 10.1016/j.abrep.2020.100335 (PMC7820913; doi:10.1016/j.abrep.2020.100335)
Supplement: Supplementary data 1 [file mmc1.docx]

**Appendix**

**Figures Depicting Moderation Results**

**Appendix A**

**
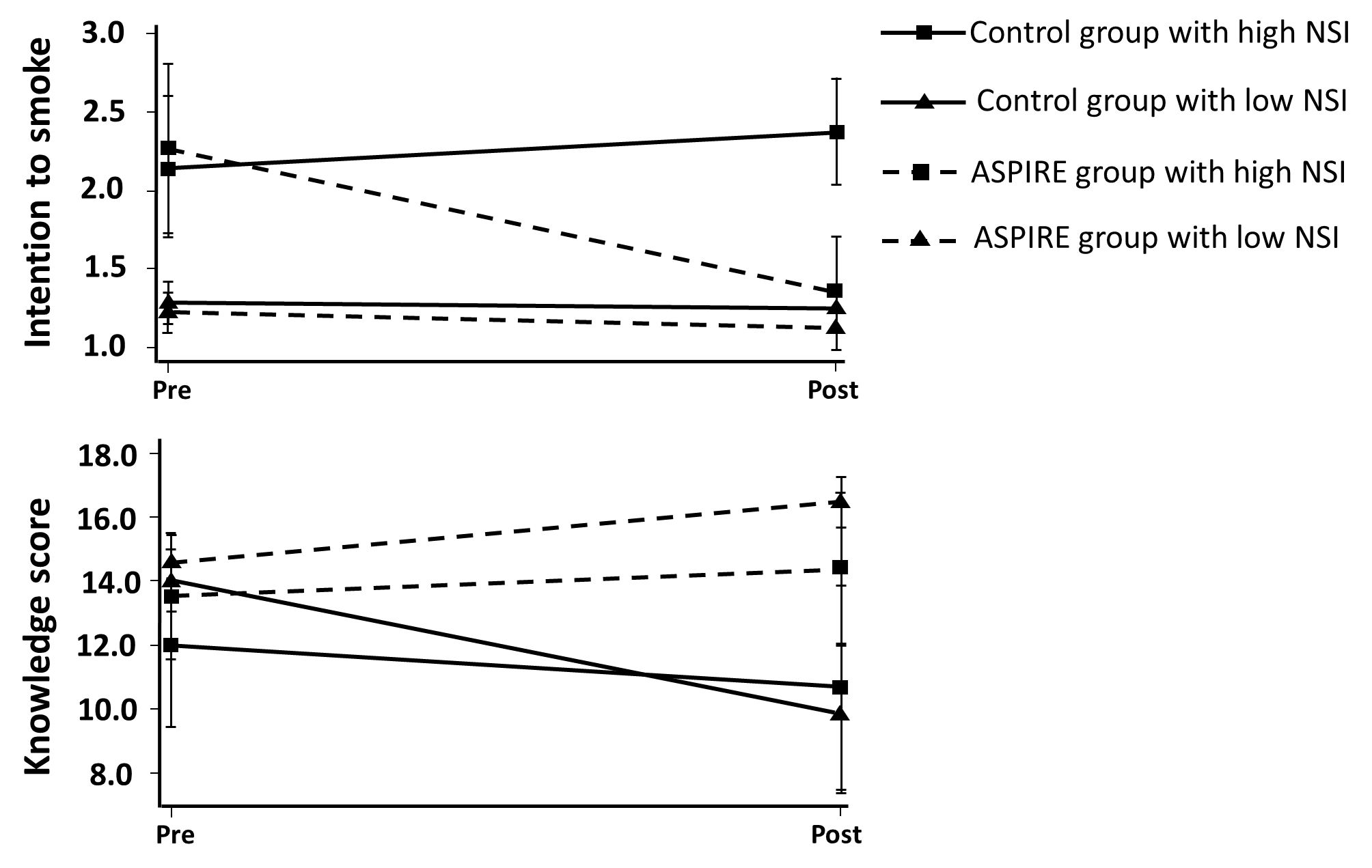
**

**Figure.** ASPIRE effect on intention to smoke and knowledge over time, by group and baseline negative social influence. NSI stands for negative social influence.

**Appendix B**


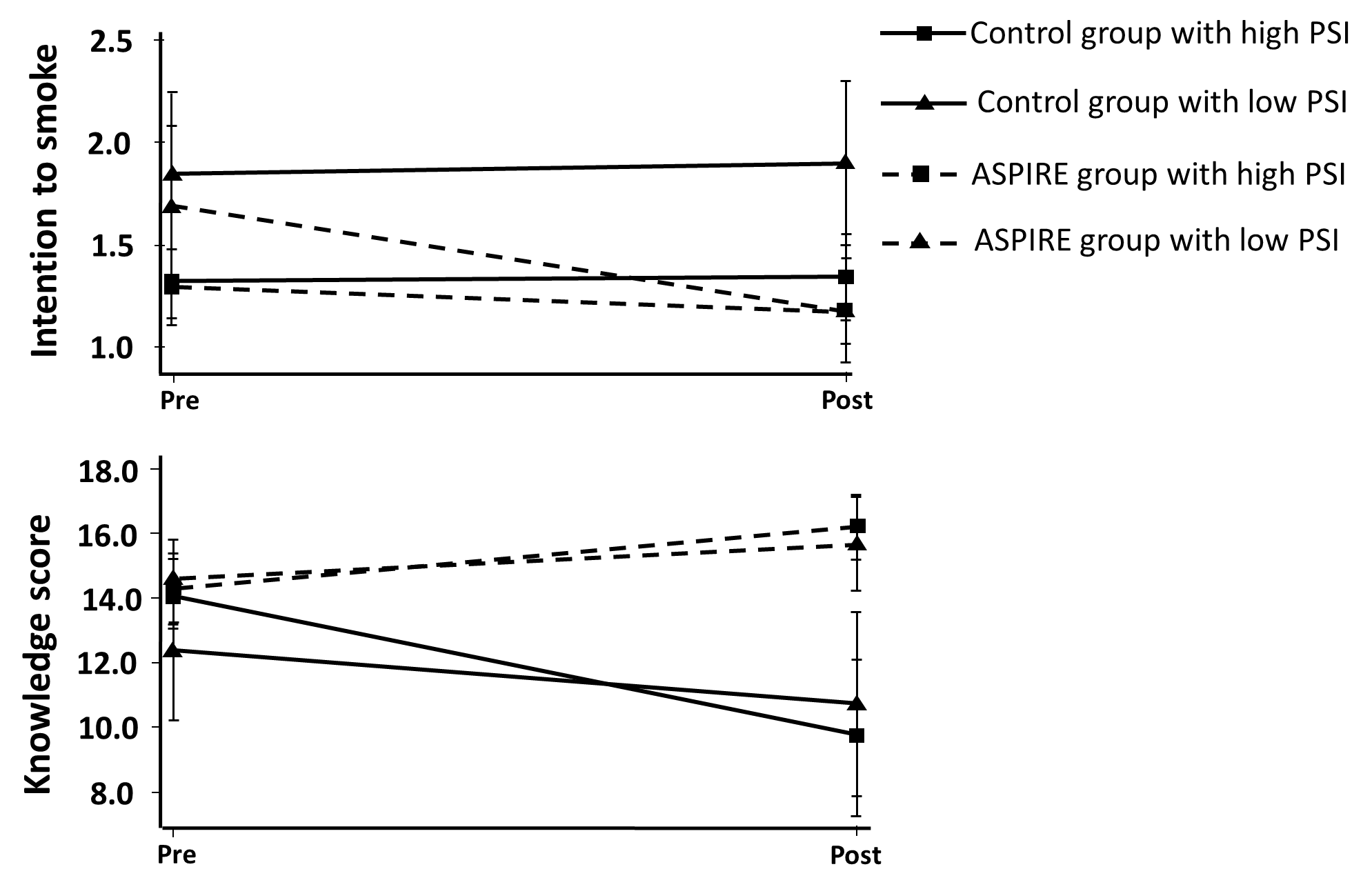


**Figure.** ASPIRE effect on intention to smoke and knowledge over time, by group and baseline positive social influence. PSI stands for positive social influence.

**Appendix C**


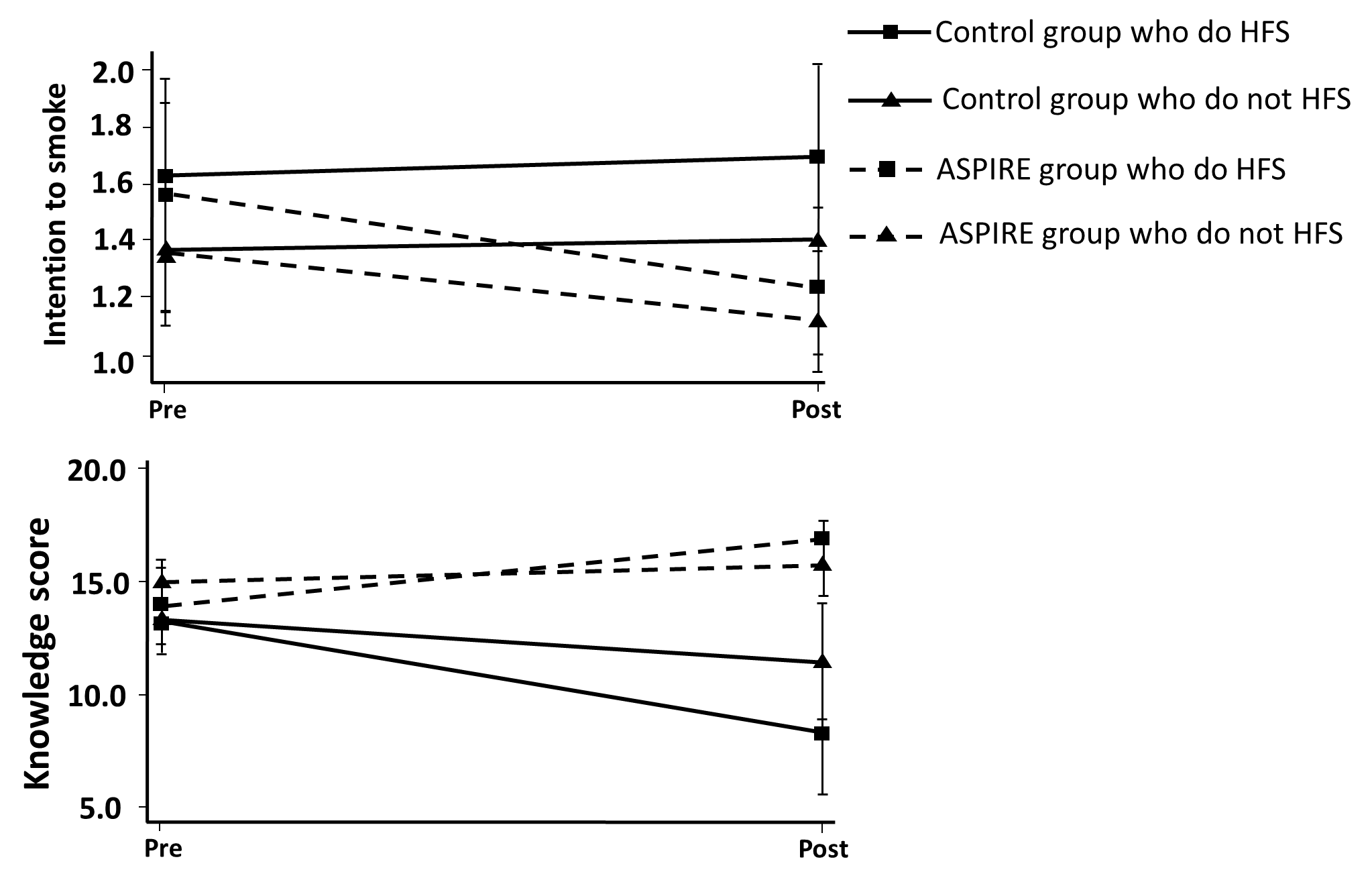


**Figure.** ASPIRE effect on intention to smoke and knowledge over time, by group and having friends who smoke. HFS stands for have friends who smoke.
